# Can exercise improve outcomes for frail haemodialysis patients?

**DOI:** 10.1093/ckj/sfae138

**Published:** 2024-05-03

**Authors:** Alice Radley, Amaryllis H Van Craenenbroeck, Kate I Stevens

**Affiliations:** NHS Greater Glasgow and Clyde Renal and Transplant Unit, Glasgow, UK; Katholieke Universiteit Leuven Universitaire Ziekenhuizen Leuven, Nephrology, Leuven, Belgium; KU Leuven, Department of Microbiology, Immunology and Transplantation, Leuven, Belgium; Glasgow Renal and Transplant Unit, Queen Elizabeth University Hospital, Glasgow, UK

End-stage kidney disease (ESKD) is associated with catabolic metabolism, systemic inflammation and sarcopenia [1]. These factors predispose frailty, a state of reduced resilience to overcome physiological stressors [2]. Frailty is not only a concern for older, comorbid patients, as 63% of haemodialysis (HD) patients <40 years of age demonstrate frailty indicators [3].

Physical activity is an effective intervention that can both prevent frailty and improve the physical performance of frail older adults [4]. It can be defined as any movement of skeletal muscle that results in energy expenditure, and includes activities of daily living (ADLs) [5]. In contrast, exercise is a structured, repetitive form of physical activity that aims to improve one or more aspects of physical fitness [5].

Current guidelines recommend 150 min of moderate intensity activity or 75 min of vigorous activity per week [6], which may prove an unrealistic and overwhelming goal for many frail HD patients. This is reflected in the low activity levels among the HD population: 44% perform no regular physical activity [7].

Physical inactivity has a detrimental effect on overall health and well-being, with sedentary HD patients having higher mortality rates and poorer quality-of-life indicators than those who report regular physical activity [8]. Inactivity causes deconditioning, with loss of muscle mass and function [9]. Regular physical activity has a protective effect on cognitive function in HD patients [10].

Physical function is also a core component of kidney transplant assessment. Clinicians commonly perceive older ESKD patients as frail, even without objective measurements of physical performance [1]. This generates disparity in access to transplantation, the treatment option with the best survival and quality of life benefits for ESKD patients, including older adults [11].

Physical performance tools that measure lower limb function predict morbidity and mortality in ESKD [12]. Walking speed, gait and standing balance can identify patients who are unlikely to benefit from kidney transplantation and can also highlight those who may benefit from ‘pre-habilitation’ (pre-hab)—physical therapy prior to transplant assessment [1].

Older adults with ESKD and those with cardiovascular comorbidities or risk factors such as smoking are least likely to perform regular physical activity [8]. HD patients cite pain, fatigue and thirst as important barriers to performing exercise [13]. Other barriers include the perception that exercise is detrimental to overall health, may worsen existing medical conditions or may compromise arteriovenous access [14].

In contrast, frail HD patients highly value functional training and are motivated by activities that focus on improving their ability to perform ADLs [15]. Intradialytic exercise can reduce community fall rates and thus prevent emergency hospitalization [15].

Exercise programs have wide-ranging benefits for HD patients, with one study reporting a 29% reduction in mortality or hospitalization among patients who completed a home-based walking program [16].

Patients who exercise during group HD sessions benefit from peer support, which improves engagement: HD patients report feeling self-conscious exercising in public [15]. Structured home exercise programs can improve cognitive function and encourage meaningful social interaction [17].

Exercise programs can be delivered safely, without adverse cardiovascular events, increased symptom burden or vascular access complications [17]. Physiotherapy supervision can build confidence among frail HD patients who are concerned about the negative impact of exercise on symptom burden [15].

Anding-Rost *et al*. [18] sought to evaluate whether exercise programs during HD positively impact patients’ physical function compared with standard HD care alone. The study population comprised 1211 prevalent, long-term HD patients (median age 65 years) with representative multimorbidity, including cardiovascular disease and diabetes, who participated in thrice weekly supervised cycling and personalized resistance exercises during HD sessions.

**Figure 1:**
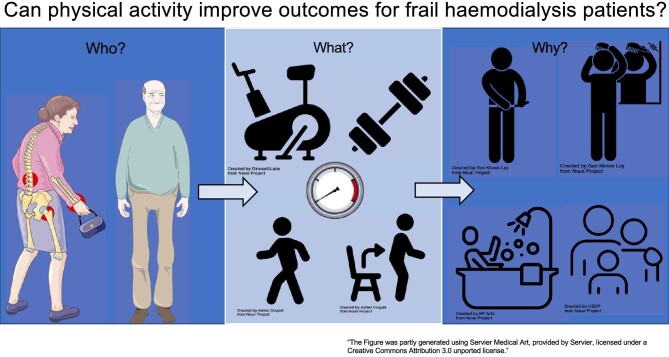
Can physical activity improve outcomes for frail haemodialysis patients?

The primary outcome was a 60-s sit to stand, a measure of lower limb strength and endurance. The exercise group improved performance during 12 months of follow-up (16.2 to 19.2 repetitions) while the control group deteriorated (16.2 to 14.7 repetitions).

The exercise group also improved in secondary outcome measures of lower limb strength and coordination and 6-min walk test, demonstrating aerobic fitness benefits. Despite these benefits, it was striking that 33% declined to participate in the exercise study, perhaps reflecting previously cited barriers to exercise.

However, this study reinforced that supervised intradialytic exercise can be delivered safely, with similar adverse event rates between the two groups and no excess mortality in the exercise group. Those participating in intradialytic exercise experienced fewer hospital admissions (1.32 versus 1.14; *P* = .02) and fewer inpatient bed days (5 versus 2; *P* = .04).

Supervised intradialytic exercise can be delivered safely and can reduce validated frailty indicators in older adults. There is a clear need to address participation of HD patients in physical activity so that they can harness the wide-ranging health and well-being benefits.

Programs should focus on achievable, patient-centred goals and prioritize activities that maximize independence and build confidence performing ADLs rather than exercise that may not be achievable or engaging for frail HD patients.

There are no robust randomized controlled trials evaluating the effects of increased physical activity in frail HD patients being considered for transplantation. To establish whether pre-hab can successfully reduce frailty indicators and promote access to transplantation, a study should be undertaken in which the intervention is focused on increasing independence with ADLs, using validated physical performance tools to measure change. This would allow us to assess whether pre-hab programs for frail HD patients are achievable, whether they reliably reduce frailty indicators and whether they improve access to transplant assessment. Further work would then be needed to evaluate whether pre-hab leads to sustained improvements in physical activity levels post-transplantation and whether it influences transplant outcomes such as graft survival and mortality.

## References

[1] Basu A . Role of physical performance assessments and need for a standardized protocol for selection of older kidney transplant candidates. Kidney Int Rep 2019;4:1666–76. 10.1016/j.ekir.2019.09.014 31844803 PMC6895582

[2] Nixon AC, Bampouras TM, Pendleton N et al. Frailty and chronic kidney disease: current evidence and continuing uncertainties. Clin Kidney J 2018;11:236–45. 10.1093/ckj/sfx134 29644065 PMC5888002

[3] Ryan L, Brown E. Supporting and maintaining the frail patient on long-term renal replacement therapy. Clin Med 2020;20:139–41. 10.7861/clinmed.2019-0416 PMC7081822 32188646

[4] British Geriatrics Society . Introduction to Frailty, Fit or Frailty Part 1. https://www.bgs.org.uk/resources/introduction-to-frailty

[5] Caspersen CJ, Powell KE, Christenson GM. Physical activity, exercise, and Physical fitness: definitions and distinctions for health-related research. Public Health Rep 1985;100:126–31. 3920711 PMC1424733

[6] World Health Organization . WHO guidelines on physical activity and sedentary behaviour. Geneva: World Health Organization, 2020. https://www.who.int/publications-detail-redirect/9789240015128 33369898

[7] Baker LA, March DS, Wilkinson TJ et al. Clinical practice guideline exercise and lifestyle in chronic kidney disease. BMC Nephrol 2022;23:75. 10.1186/s12882-021-02618-1 35193515 PMC8862368

[8] Tentori F, Elder SJ, Thumma J et al. Physical exercise among participants in the Dialysis Outcomes and Practice Patterns Study (DOPPS): correlates and associated outcomes. Nephrol Dial Transplant 2010;25:3050–62. 10.1093/ndt/gfq138 20392706

[9] Manfredini F, Lamberti N, Malagoni AM et al. The role of deconditioning in the end-stage renal disease myopathy: physical exercise improves altered resting muscle oxygen consumption. Am J Nephrol 2015;41:329–36. 10.1159/000431339 26067552

[10] Baggetta R, D'Arrigo G, Torino C et al. Effect of a home based, low intensity, physical exercise program in older adults dialysis patients: a secondary analysis of the EXCITE trial. BMC Geriatr 2018;18:248. 10.1186/s12877-018-0938-5 30342464 PMC6196029

[11] Pinter J, Hanson CS, Craig JC et al. ‘I feel stronger and younger all the time’—perspectives of elderly kidney transplant recipients: thematic synthesis of qualitative research. Nephrol Dial Transplant 2016;31:1531–40. 10.1093/ndt/gfv463 27333617

[12] Nixon AC, Bampouras TM, Pendleton N et al. Diagnostic accuracy of frailty screening methods in advanced chronic kidney disease. Nephron 2019;141:147–55. 10.1159/000494223 30554199

[13] Lightfoot CJ, Wilkinson TJ, Song Y et al. Perceptions of exercise benefits and barriers: the influence on physical activity behaviour in individuals undergoing haemodialysis and peritoneal dialysis. J Nephrol 2021;34:1961–71. 10.1007/s40620-021-01024-y 33770396 PMC8610943

[14] Jayaseelan G, Bennett PN, Bradshaw W et al. Exercise benefits and barriers: the perceptions of people receiving hemodialysis. Nephrol Nurs J 2018;45:185–91. 30303639

[15] Young HML, March DS, Highton PJ et al. Exercise for people living with frailty and receiving haemodialysis: a mixed-methods randomised controlled feasibility study. BMJ Open 2020;10:e041227. 10.1136/bmjopen-2020-041227 PMC7640592 33148767

[16] Mallamaci F, D'Arrigo G, Tripepi G et al. Long-term effect of physical exercise on the risk for hospitalization and death in dialysis patients. Clin J Am Soc Nephrol 2022;17:1176–82. 10.2215/CJN.03160322 35878932 PMC9435990

[17] Manfredini F, Mallamaci F, D'Arrigo G et al. Exercise in patients on dialysis: a multicenter, randomized clinical trial. J Am Soc Nephrol 2017;28:1259–68. 10.1681/ASN.2016030378 27909047 PMC5373448

[18] Anding-Rost K, von Gersdorff G, von Korn P et al. Exercise during hemodialysis in patients with chronic kidney failure. NEJM Evid 2023;2:EVIDoa2300057. 10.1056/EVIDoa2300057 38320198

